# Supplementary material for: Controllable cyanation of carbon-hydrogen bonds by zeolite crystals over manganese oxide catalyst
Source: Nat Commun. 2017 May 15;8:15240. doi: 10.1038/ncomms15240 (PMC5440663; doi:10.1038/ncomms15240)
Supplement: Supplementary Information — Supplementary figures, supplementary tables, supplementary notes, supplementary methods and supplementary references. [file ncomms15240-s1.pdf]

Supplementary Methods

Chemicals

All chemicals were of analytical grade and used as purchased without further purification.

Synthesis of referenced catalysts

Synthesis of Au/ZSM-5. As a typical run, 1 g of ZSM-5 support (Si/Al ratio at 35) were added into a 200 mL of chloroauric acid aqueous solution containing 10 mg of Au and 0.24 g of urea. After stirring at 90 °C for 4 h in a closed reactor to keep away from light, removing the water under vacuum, drying at 100 °C for 12 h, and calcining at 400 °C in air for 2 h, the Au/ZSM-5 sample was obtained. By analysis of inductively coupled plasma spectrometer (ICP), the Au loading on Au/ZSM-5 was at 0.82 *wt*%.

Synthesis of Pd/CN. As a typical run, 1 g of chitosan and 0.2 g of borax was added into 24.4 g of acetic aqueous solution (2.0 *wt*%) and stirred for 3 h at room temperature. Then the mixture was transferred into an autoclave and heated at 200 °C for 10 h. The as-synthesized carbon nitride support was collected by filtrating and calcining at 550 °C for 2 h in N_2. For loading the Pd nanoparticles, 0.5 of the carbon nitride support was dispersed in the solution containing water (200 g), $PdCl_2$ (7 mg of Pd), and urea (87 mg). After stirring at 90 °C for 5 h, cooling to room temperature, adding 6 ml of $NaBH_4$ solution (10 mg/ml), and stirring for another 6 h. The solid was filtrated, washed with large amount of water, and died for overnight to obtain the final catalyst with Pd loading at 1.1 *wt*%.

FT-IR spectra of adsorbed probing molecules

In a typical experiment, 30 mg of the powder sample was dried in vacuum at 150 ℃ for 12 h to remove the water species, followed by addition of 2,4-quinolinediol solution (100 mg of 2,4-quinolinediol in 10 ml of ethyl acetate). After stirring for 30

min at room temperature, the solid powder was separated by centrifugation, and dried at 120 ℃ under vacuum to remove the ethyl acetate. Then, the FTIR spectra were recorded using a Bruker 66V FTIR spectrometer at room temperature. Each sample was recorded with 32 scans with resolution of 2 cm^{-1}.

Oxidation of probing molecules

The oxidation of probing molecules of 3,5-dimethylbenzylalcohol and benzyl alcohol was carried out in a conventional continuous-flow fixed-bed quartz vertical reactor (length of 400 mm and diameter of 6 mm). The temperature was controlled at 220 ℃ by an oven. Pure oxygen was used as oxidant. 0.2 g of catalyst (20-40 mesh) diluted with 0.2 g of quartz sands (20-40 mesh) was located in the reactor. The oxidation occurred by inletting probing molecules dissolved in *p*-xylene with a pump (0.120 ml/h) and the flow rate of oxygen was controlled by a flow instrument (6.4 ml/min). The reactants and products were collected by cold trap and then analyzed by a Shimadzu GC-2014 chromatograph equipped with FID detector.

Catalytic tests in hydration of benzonitrile

The hydration of benzonitrile was performed in the same reactor except using N_2 instead of O_2. The typical reaction conditions were as follows: 100 mg of catalyst, 28 mmol of toluene or a mixture of 25 mmol of toluene and 3 mmol of water as solvent, 3 mmol of benzonitrile, 170 °C, 4 h, 2.0 MPa of N_2, and *n*-dodecane was used as internal standard.

Carbon balance of the reaction system

The carbon balance of the system before and after reaction was based on the number of carbon atoms in all the detectable compounds in GC analysis using *n*-dodecane as internal standard. In most reactions, the GC detectable products are benzonitrile, benzamide, benzaldehyde, benzyl alcohol, and benzonic acid. The value of carbon balance was calculated by the following equation:

$$C\% = \frac{m_0 \times 7 + m_1 \times n_1 + m_2 \times n_2 + m_3 \times n_3 + \ldots + m_x \times n_x}{m_t \times 7} \times 100\%$$

Where C% is the value of carbon balance, m_0 is the moles of toluene in the reactor after reaction; 7 is the number of carbon atoms in one toluene molecule; m_1 (m_2, m_3…..m_x) is the moles of product 1 (product 2, 3……x) in the reactor after reaction; n_1 (n_2, n_3…..n_x) is the number of carbon atoms in one molecule of product 1 (product 2, 3……x); m_t is the mole of toluene substrate before reaction.

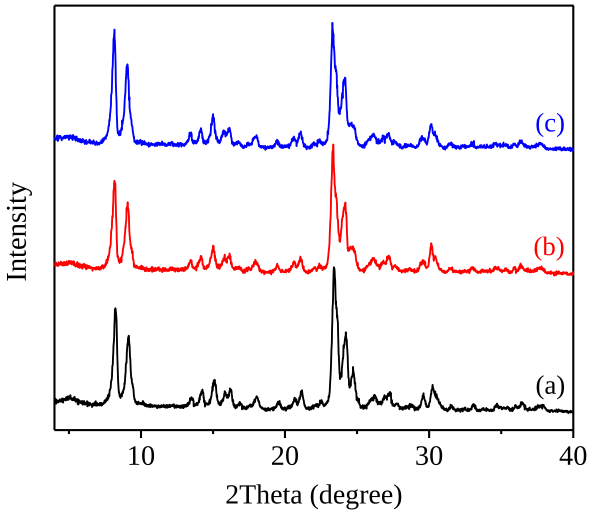

Supplementary Figure 1. XRD patterns of (a) S-1, (b) MnO_x/S-1, and (c) MnO_x@S-1. All samples exhibit XRD peaks associated with typical MFI zeolite structure. In addition, it is difficult to observe the peaks related to MnO_x crystals, indicating the high dispersion of the MnO_x species.

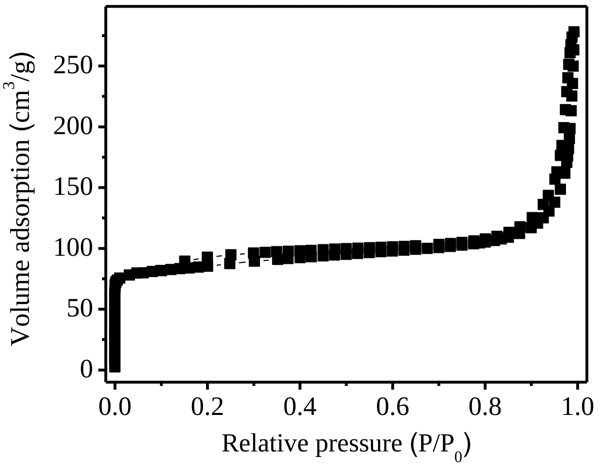

Supplementary Figure 2. N_2 sorption isotherms of MnO_x@S-1. MnO_x@S-1 gives high surface area of 375 m^2/g and microporous size of 0.54 nm.

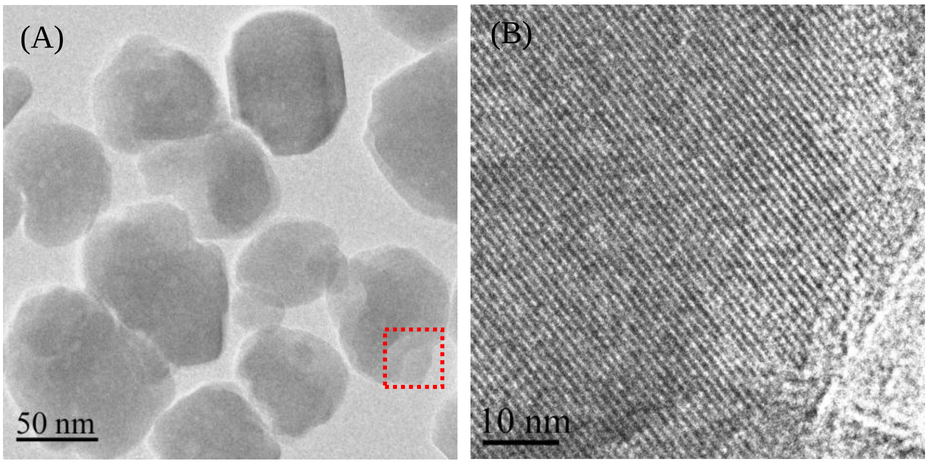

Supplementary Figure 3. (A) TEM image of S-1 zeolite; (B) Enlarged view of the red square in **A**.

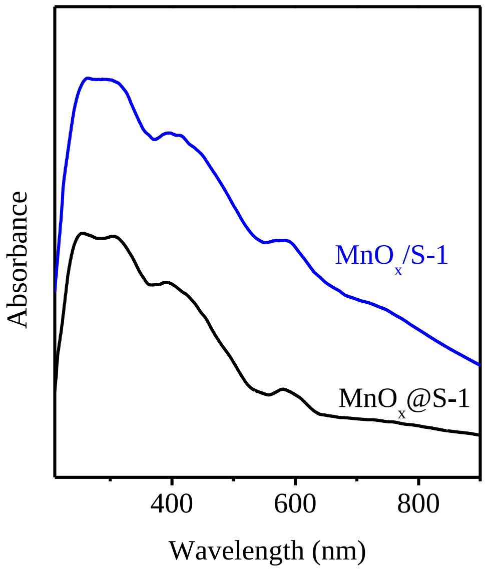

Supplementary Figure 4. UV-Visible spectra of MnO_x/S-1 and MnO_x@S-1 samples.

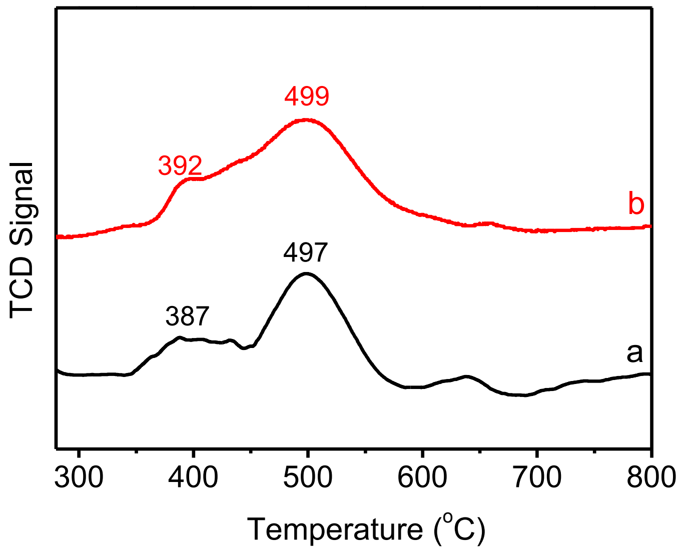

Supplementary Figure 5. O_2-TPD profiles of (a) MnO_x@S-1 and (b) MnO_x/S-1.

Supplementary Note 1

The oxygen desorption at low temperature (< 400 ℃) is ascribed to the adsorbed oxygen, while oxygen desorption at high temperature (> 400 ℃) is attributed to the lattice oxygen. Both samples exhibit similar desorption peaks assigned to adsorbed oxygen and lattice oxygen at 387-392 and 497-499 ℃, respectively.[1-3] These results suggest their similar oxygen species.

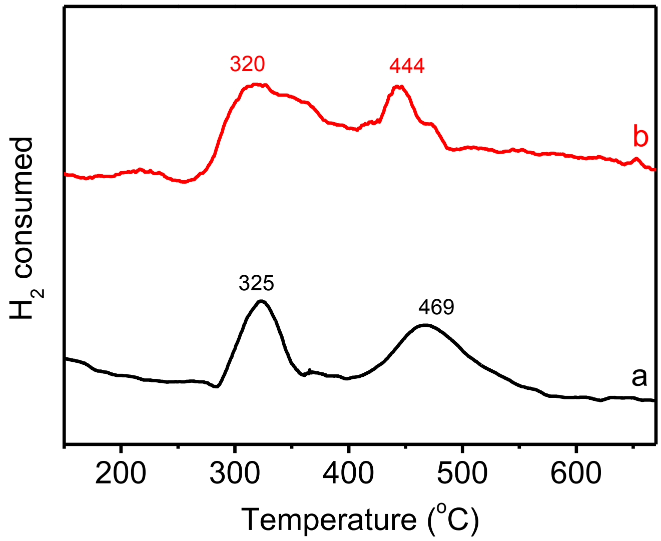

Supplementary Figure 6. H_2-TPR profiles of (a) MnO_x@S-1 and (b) MnO_x/S-1.

Supplementary Note 2

Both samples give similar peaks at 320-325 and 444-469 ℃, which are assigned to the reduction of Mn^{4+} ($Mn^{4+} \rightarrow Mn^{3+}$) and Mn^{3+} ($Mn^{3+} \rightarrow Mn^{2+}$), respectively.[2,3]

Supplementary Table 1. Oxidative cyanation of toluene over the MnO_x@S-1 catalyst in various solvents.

Entry	Solvent	TOF (h^{-1})[†]	Yield (%)[‡]	
			Benzonitrile	Benzamide
1	Toluene	32.5	88.6	<1.0
2	*N*,*N*-Dimethylformamide	22.2	14.3	18.9
3	Tetrahydrofuran	2.3	2.1	1.1
4	*t*-Butyl alcohol	<1.0	--[⊥]	--[⊥]
5	CH_2Cl_2	2.2	3.3	--[⊥]
6	Water	9.7	--[⊥]	--[⊥]

Reaction conditions: 35 mg of catalyst, 0.5 mmol of urea, 160 °C, 6 h, 1.5 MPa of O_2, 5 mmol of toluene, and 2 ml of solvent. In the case of toluene, 28 mmol of toluene was used as both substrate and solvent.

[†]The TOFs were calculated based on the total amount of metal atoms in the reaction system at a reaction time of 1 h.

[‡]The yields were determined based on the amount of urea, and *n*-dodecane was used as internal standard.

[⊥]Undetectable.

Supplementary Table 2. The yield of nitrogen-free by-products in the oxidative cyanation reactions.

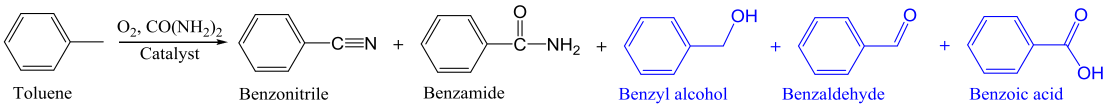

Entry	Catalyst	Yield (%)†		
		Benzyl alcohol	Benzaldehyde	Benzonic acid
1	S-1	--	--	--
2	MnO_x/S-1	2.4	18.5	5.3
3	MnO_x/SiO_2	3.0	16.1	2.2
4	MnO_x@S-1	4.4	9.0	9.3
5	MnO_x@S-1, in air$^{\perp}$	7.9	12.2	<1.0
6	$Mn(CH_3COO)_2$	5.5	65.7	92.3
7	$KMnO_4$	<1.0	<1.0	<1.0

The reaction conditions are the same to those in Table 1.

† For comparison with the yields of benzonitrile, the yields of these nitrogen-free by-products were determined based on the amount of nitrogen in the feed, *n*-dodecane was used as internal standard. Yield = (amount of product) / (amount of nitrogen in the feed)*100%

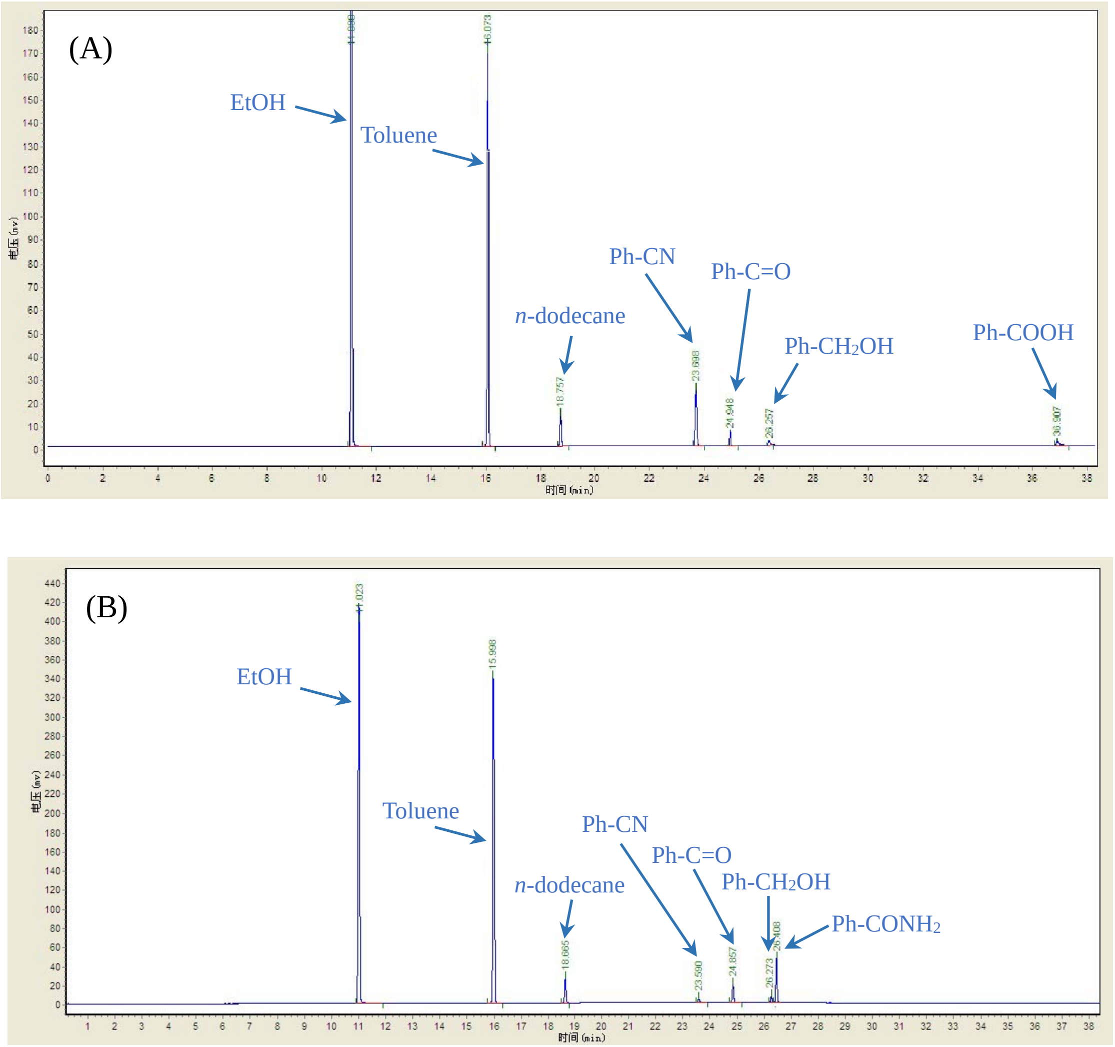

Supplementary Figure 7. GC traces for the oxidative cyanation of toluene over (A) MnO_x@S-1 and (B) MnO_x/S-1 catalysts, corresponding to the experiments in entries 2 and 3 in Supplementary Table 4, respectively. The EtOH was added to dilute the mixture after reaction.

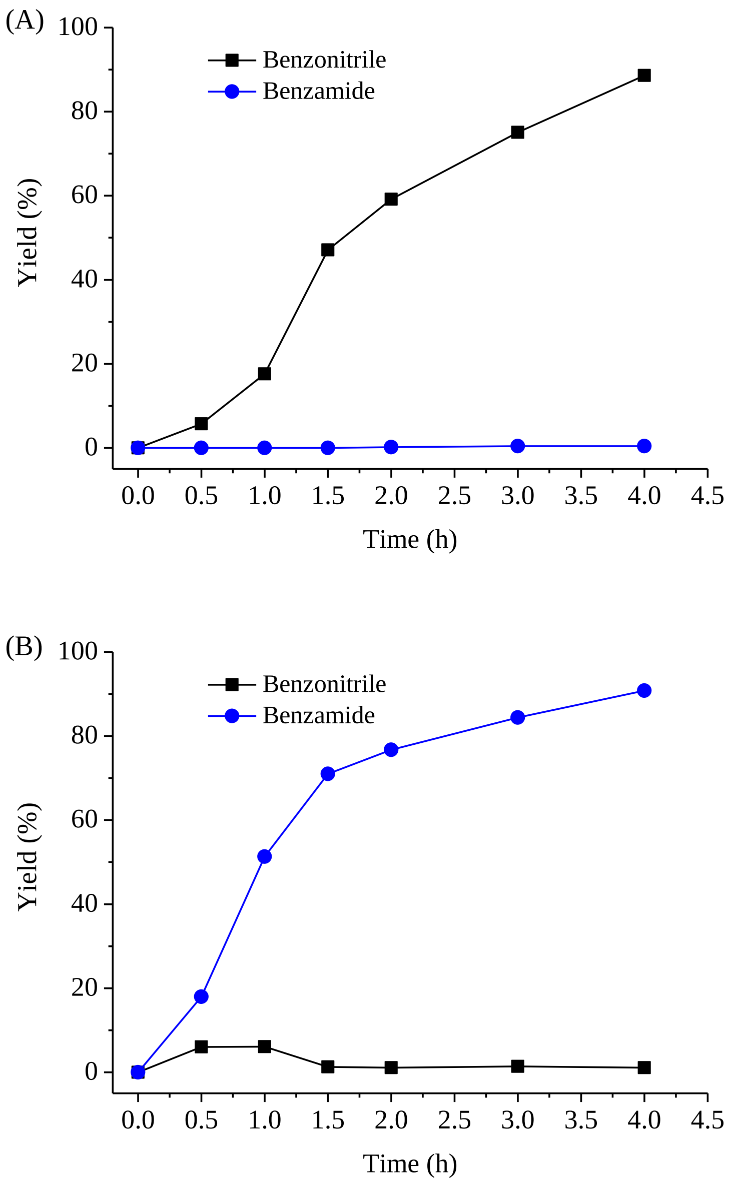

Supplementary Figure 8. Dependences of benzonitrile and benzamide yields on time over (A) MnO_x@S-1 and (B) MnO_x/S-1 catalysts.

Supplementary Table 3. Catalytic oxidative cyanation of various hydrocarbons over MnO_x@S-1 using gaseous ammonia.

$$R{-}CH_3 \xrightarrow[\text{Catalyst}]{O_2,\ NH_3} R{-}CN$$

Yields of various nitriles (%)		
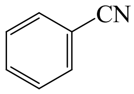	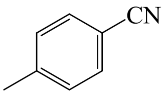	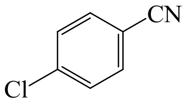
78.4	66.4	64.7
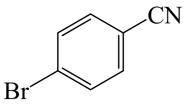	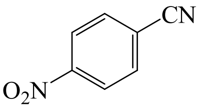	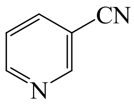
67.3	71.3	84.1
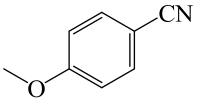	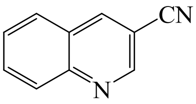	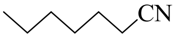
82.2	56.7	16.6

Reaction conditions: 35 mg of catalyst, 28 mmol of substrate, 1.0 mmol of gaseous ammonia, 160 °C, 4 h, 1.5 MPa of O_2, the yields were determined based on the amount of nitrogen in the feed, *n*-dodecane was used as internal standard.

Supplementary Table 4. Oxidative cyanation of toluene over the MnO_x@S-1 catalyst using various nitrogen sources.

Entry	N sources	TOF (h^{-1})†	Yield (%)‡	
			Benzonitrile	Benzamide
1	urea	32.5	88.6	<1.0
2	urea†	19.9	69.7	2.6
3	urea†‡	26.3	5.9	76.0
4	ammonium hydroxide	40.0	62.0	28.0
5	$(NH_4)_2CO_3$	28.9	31.1	3.3
6	NH_4HCO_3	33.1	45.6	4.9
7	urea + K_2CO_3⊥	32.3	80.1	<1.0
8	urea + K_2CO_3&	35.4	47.3	5.9
9	urea + CH_3COOK#	29.8	85.9	<1.0
10	urea + $CO_2$$	32.2	85.0	<1.0

Reaction conditions: 35 mg of catalyst, 28 mmol of toluene, 0.5 mmol of urea or other sources with equal amount of nitrogen , 160 °C, 4 h, and 1.5 MPa of O_2. The TOFs were calculated based on the total amount of metal atoms in the reaction system at a reaction time of 0.5 h. The yields were determined based on the amount of nitrogen atom, and *n*-dodecane was used as internal standard.

† 80 mg of catalyst, 1.5 mmol of urea, and reaction time at 9 h.

‡ MnO_x/S-1 catalyst was used.

⊥ molar ratio of urea/K_2CO_3 at 5.

& molar ratio of urea/K_2CO_3 at 1.

molar ratio of urea/KCl at 2.5.

$ 0.5 mmol of CO_2.

Supplementary Note 3

To investigate the role of carbonate, we artificially added K_2CO_3 into the reactor with

urea as nitrogen sources. When a small amount of K_2CO_3 (molar ratio of urea/K_2CO_3 at 5, entry 7 in Supplementary Table 4) was added in the feed, the MnO_x@S-1 catalyst exhibit lower benzonitrile yield at 80.1% than that of the K_2CO_3-free system (88.6%, entry 1). Further increase the amount of K_2CO_3 (molar ratio of urea/K_2CO_3 at 1, entry 8 in Supplementary Table 4) lead to the significant decrease of benzonitrile yield at 47.3% with the formation of a large amount of benzoic acid (entry 4, Supplementary Table 5). The obvious change in product selectivity is undetectable with the addition of KCl or CO_2 instead of K_2CO_3. Based on these results, it is suggested that the carbonyl group could promote the direct oxidation into benzoic acid, thus hindering the cyanation to benzonitrile.

Supplementary Table 5. The yield of nitrogen-free by-products in the oxidative cyanation of toluene over the MnO_x@S-1 catalyst using various nitrogen sources.

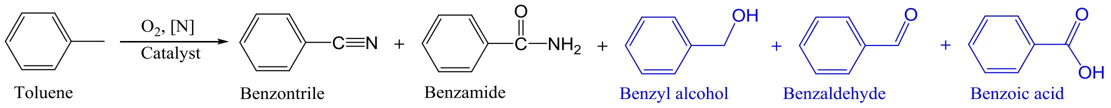

Entry	N sources	Yield (%)†		
		Benzyl alcohol	Benzaldehyde	Benzonic acid
1	urea	4.4	9.0	9.3
2	$(NH_4)_2CO_3$	<1.0	9.5	38.9
3	urea + K_2CO_3⊥	2.0	16.1	17.2
4	urea + K_2CO_3&	<1.0	4.2	33.3
5	urea + KCl#	<1.0	13.8	11.0
6	urea + $CO_2$$	3.5	8.1	7.3
7	ammonium hydroxide	5.0	10.7	9.9

The reaction conditions are the same to those in Supplementary Table 4.

† For comparison with the yields of benzontrile, the yields of these nitrogen-free by-products were based on the amount of nitrogen in the feed, *n*-dodecane was used as an internal standard. Yield = (amount of product) / (amount of nitrogen in the feed)*100%.

⊥ molar ratio of urea/K_2CO_3 at 5.

& molar ratio of urea/K_2CO_3 at 1.

molar ratio of urea/KCl at 2.5.

$ 0.5 mmol of CO_2.

Supplementary Table 6. Catalytic oxidative amidation of various hydrocarbons over MnO_x/SiO_2 and MnO_x/S-1.

Catalyst	Yields of various amides (%)		
	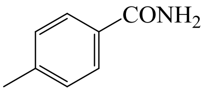	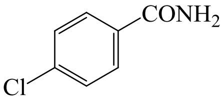	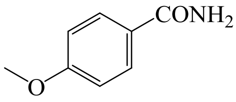
MnO_x/SiO_2	60.0	50.5	65.1
MnO_x/S-1	57.9	54.4	65.9
	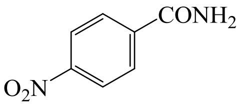	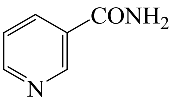	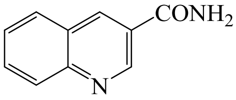
MnO_x/SiO_2	78.0	66.6	81.4
MnO_x/S-1	71.5	72.0	73.3
	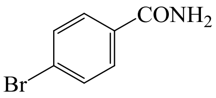	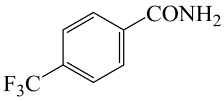	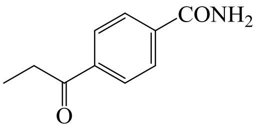
MnO_x/SiO_2	56.1	48.3	82.0
	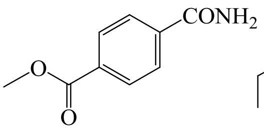	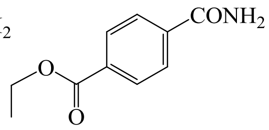	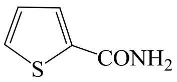
MnO_x/SiO_2	77.2	78.4	50.1

Reaction conditions: 35 mg of catalyst, 28 mmol of substrate, 0.5 mmol of urea, 160 °C, 4 h, 1.5 MPa of O_2, the yields based on the amount of nitrogen in the feed, and *n*-dodecane as an internal standard.

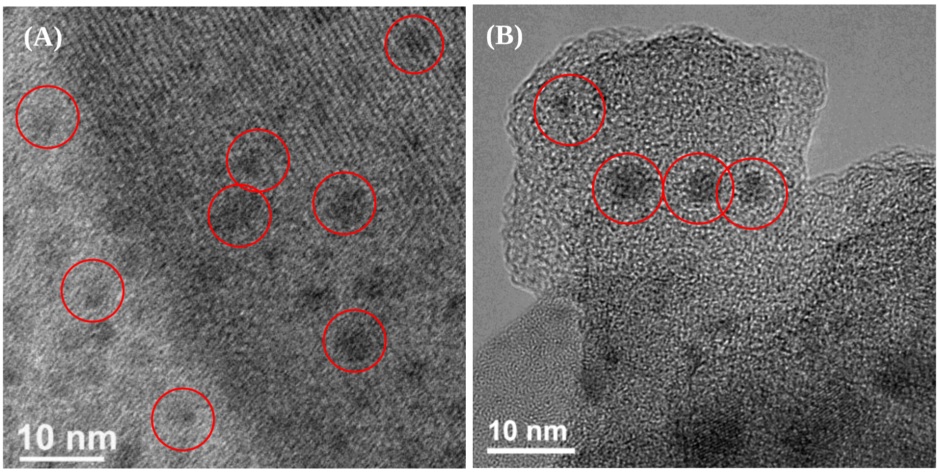

Supplementary Figure 9. High-resolution TEM images of (A) Au/ZSM-5 and (B) Pd/CN. The red cycles highlight part of the Au or Pd nanoparticles.

Supplementary Table 7. Catalytic oxidative cyanation of *n*-hexane and *n*-octane over verious reference catalysts.

Entry	Catalyst	*n*-hexane	*n*-octane
		Yield of hexanenitrile (%)	Yield of octanenitrile (%)
1	Au/ZSM-5	0.8	0.3
2	Pd/CN	--†	--†
3	$CoCl_2$	1.0	--†
4	$Co(CH_3COO)_2$	1.4	0.9

Reaction conditions: 35 mg of catalyst, 28 mmol of substrate, 0.5 mmol of urea, 185 °C, 4 h, 3.5 MPa of O_2, the yields based on the amount of nitrogen in the feed, and *n*-dodecane as an internal standard.

† Undetectable.

Supplementary Table 8. Catalytic oxidative cyanation of *n*-hexane and *n*-octane over MnO_x@S-1 under various conditions.

Entry	Reaction conditions	*n*-hexane		*n*-octane	
		Yield of hexanenitrile (%)	Carbon balance (%)	Yield of octanenitrile (%)	Carbon balance (%)
1	80 mg of catalyst, 8 h	19.2	93.2	6.8	97.9
2	160 mg of catalyst, 8 h	19.9	80.1	8.9	93.8
3	80 mg of catalyst, 20 h	23.4	65.5	10.2	70.2
4	80 mg of catalyst, 8 h, TBHP initiator †	30.3	91.5	11.9	97.0

Reaction conditions: 28 mmol of substrate, 0.5 mmol of urea, 185 °C, 3.5 MPa of O_2, the yields based on the amount of nitrogen in the feed, *n*-dodecane as an internal standard.

† 15 mg of *tert*-butyl hydroperoxide (TBHP, 50% in *n*-dodecane) added as initiator, diphenyl used as internal standard.

Supplementary Note 4

Increasing the amount of MnO_x@S-1 (entry 2) improved the yield of nitrile products very slightly. Prolonging the reaction time (entry 3) displays a significant enhancement of nitrile yields but being accompanied by decreased carbon balance values. This phenomenon is reasonably assigned to the side reaction of over oxidation—translating hydrocarbons to carbon dioxide (detected by gas chromatography with thermal conductivity detector), which is often observed in the

oxidation of hydrocarbons.[4] Furthermore, we also attempted to enhance the catalysis by the initiator (*e.g.* TBHP). It has been well documented that the addition of a small amount of initiator can effectively improve the overall catalytic performances, and this method has been widely used in many oxidation system where oxygen is used as a major oxidant.[5-7] Interestingly, by adding a small amount of TBHP as initiator in the reaction system, it is found that the MnO_x@S-1 shows remarkably enhanced yields of hexanenitrile and octanenitrile at 30.3 and 11.9%, and the carbon balance values were well maintained, demonstrating the positive role of TBHP in these reactions.

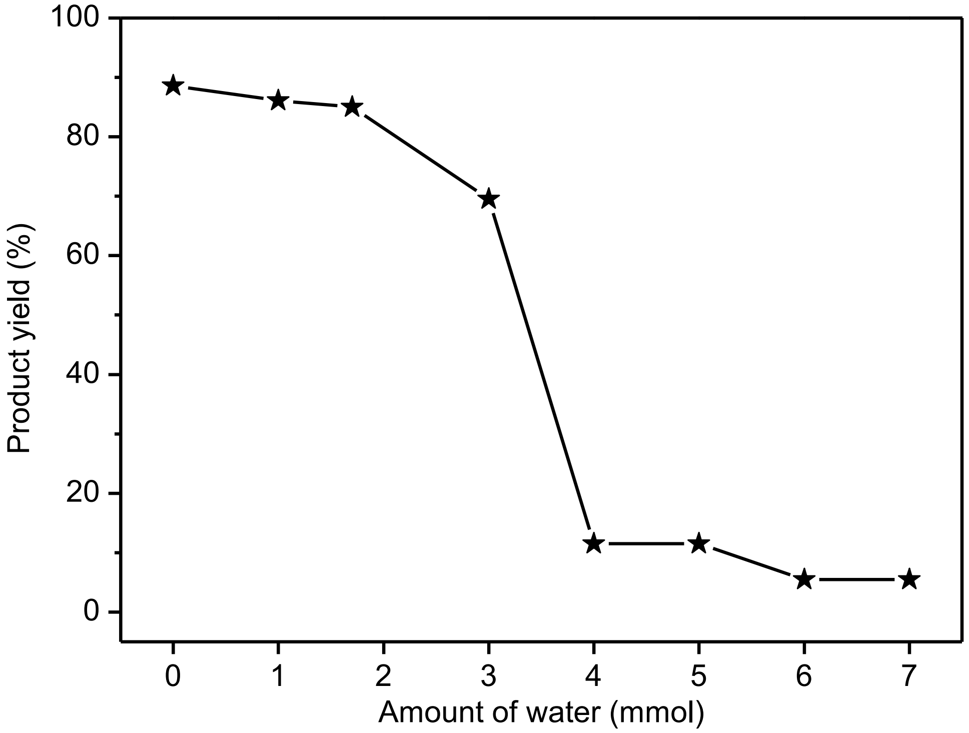

Supplementary Figure 10. The yields of benzonitrile over MnO_x@S-1 catalyst with addition of different amount of water in the reaction system. The reaction conditions are the same to those in Table 1.

Supplementary Table 9. Hydration of benzonitrile to benzamide over the MnO_x/S-1, MnO_x/SiO_2, and MnO_x@S-1 catalysts under different reaction conditions.

Entry	Catalyst	Solvent	Conv. (%)	Yield (%)
1	MnO_x/S-1	Toluene	4.8	4.8
2	MnO_x/SiO_2	Toluene	5.5	5.5
3	MnO_x@S-1	Toluene	<1.0	<1.0
4	MnO_x/S-1	Toluene + water	19.4	19.2
5	MnO_x/SiO_2	Toluene + water	15.0	13.9
6	MnO_x@S-1	Toluene + water	2.1	2.1

Reaction conditions: 80 mg of catalyst, 28 mmol of toluene or a mixture of toluene (26 mmol) and water (1.7 mmol) as solvent, 0.8 mmol of benzonitrile, 150 °C, 2 h, and 1.0 MPa of N_2. The products were analyzed by GC using *n*-dodecane as internal standard.

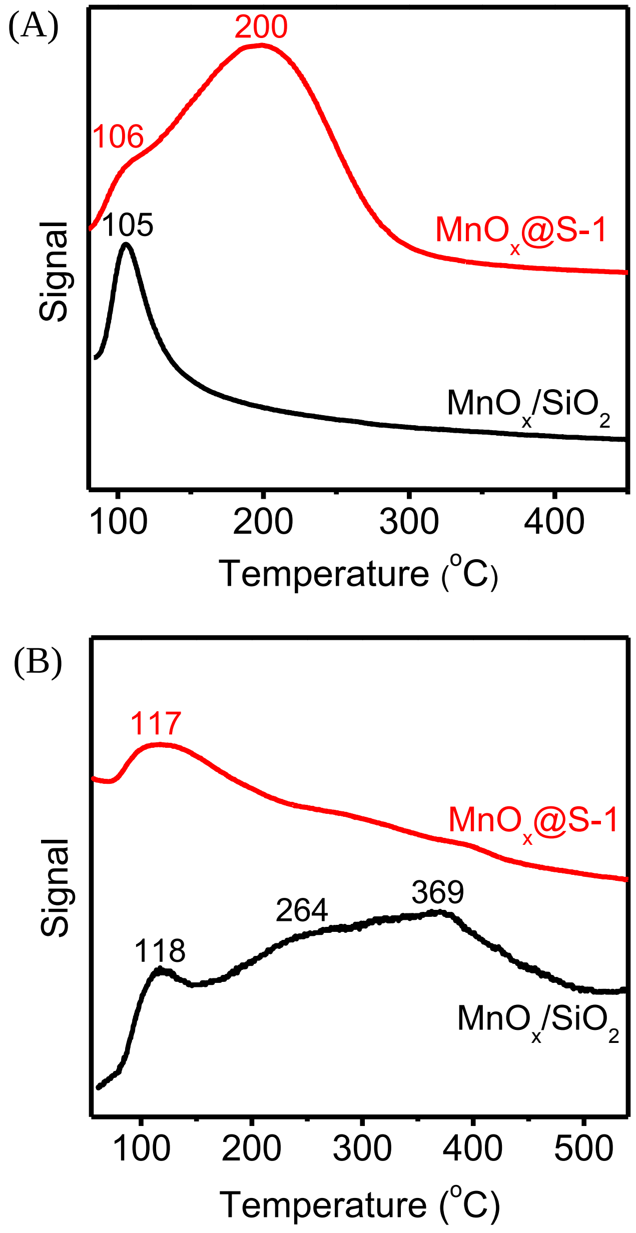

Supplementary Figure 11. TPD of (A) toluene and (B) water from MnO_x@S-1 and MnO_x/SiO_2.

Conventional multiple routes:

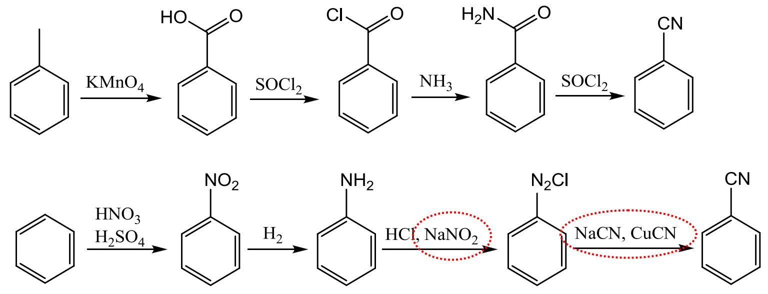

One-pot oxidative cyanation (this work):

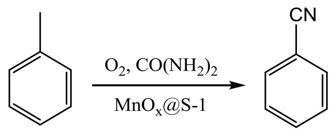

Supplementary Figure 12. Various routes for producing benzonitrile from toluene or benzene.

Supplementary Table 10. Catalytic oxidative cyanation of toluene and 1,3,5-trimethylbenzene over MnO_x@S-1 catalyst.

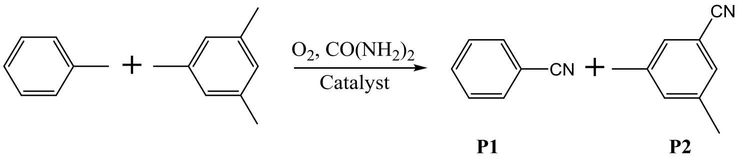

Catalyst	Product	Yield (%)†	Yield (%)‡
MnO_x@S-1	P1	85.0	87.8*
	P2	--	--
MnO_x/S-1	P1	4.2	4.5
	P2	47.1	49.5

† In the tests using mixture of toluene and 1,3,5-trimethylbenzene as substrate.

‡ In the individual test using toluene or 1,3,5-trimethylbenzene as substrate.

* Note that this yield is from an independent catalytic test from that of entry 4 in Table 1. The results from two independent experiments are well consistent.

Supplementary Table 11. Catalytic oxidative cyanation of *o*-, *m*-, *p*-xylene over MnO_x@S-1 catalyst.

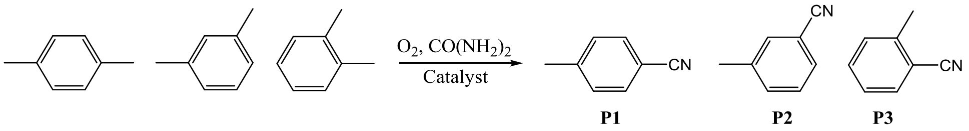

Catalyst	Product	Yield (%)†
MnO_x@S-1	P1	65.0
	P2	<1.0
	P3	2.9

† In the tests using mixture of *o*-, *m*-, and *p*-xylene with molar ratio at 1 as substrate.

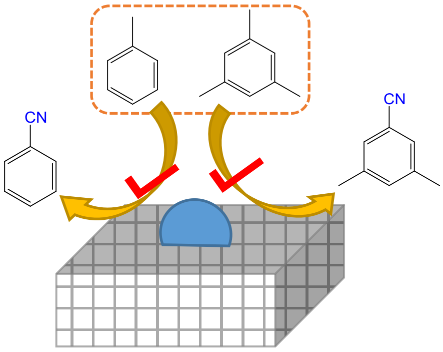

Supplementary Figure 13. Scheme describing that the MnO_x/S-1 is active for the oxidative cyanation of both toluene and 1,3,5-trimethylbenzene, due to the lack of shape selectivity.

Supplementary References

1. Liu, Y., Dai, H., Du, Y., Deng, J., Zhang, L., Zhao, Z., Au, C. T. Controlled preparation and high catalytic performance of three-dimensionally ordered macroporous $LaMnO_3$ with nanovoid skeletons for the combustion of toluene. *J. Catal.* **287**, 149-160 (2005).
2. Liu, Y., Dai, H., Deng, J., Du, Y., Li, X., Zhao, Z., Wang, Y., Gao, B., Yang, H., Guo, G. In situ poly(methyl methacrylate)-templating generation and excellent catalytic performance of MnOx/3DOM $LaMnO_3$ for the combustion of toluene and methanol. *Appl. Catal. B* **140-141**, 493-505 (2013).
3. Si, W., Wang, Y., Peng, Y., Li, J. Selective Dissolution of A-site cations in ABO_3 perovskites: A new path to high-performance catalysts. *Angew. Chem. Int. Ed.* **54**, 7954-7957 (2015).
4. Zhang, P., Lu, H., Zhou, Y., Zhang, L., Wu, Z., Yang, S., Shi, H., Zhu, Q., Chen, Y., Dai, S. Mesoporous $MnCeO_x$ solid solutions for low temperature and selective oxidation of hydrocarbons. *Nat. Commun.* **6**, 8446 (2015).
5. Hughes, M. D., Xu, Y. J., Jenkins, P., McMorn, P., Landon, P., Enache, D. I., Carley, A. F., Attard, G. A., Hutchings, G. J., King, F., Stitt, E. H., Johnston, P., Griffin, K., Kiely, C. J. *Nature* **437**, 1132-1135 (2005).
6. Zhao, R., Ji, D., Lv, G., Qian, G., Yan, L., Wang, X., Suo, J. A highly efficient oxidation of cyclohexane over Au/ZSM-5 molecular sieve catalyst with oxygen as oxidant. *Chem. Comm.* **7**, 904-905 (2004).
7. Wang, L., Zhu, Y., Wang, J.-Q., Liu, F., Huang, J., Meng, X., Basset, J. M., Han, Y., Xiao, F.-S. Two-dimensional gold nanostructures with high activity for selective oxidation of carbon-hydrogen bonds. *Nat. Commun.* **6**, 6957 (2015).
